# Epidemiology of subpatent *Plasmodium falciparum* infection: implications for detection of hotspots with imperfect diagnostics

**DOI:** 10.1186/1475-2875-12-221

**Published:** 2013-07-01

**Authors:** Jacklin F Mosha, Hugh JW Sturrock, Bryan Greenhouse, Brian Greenwood, Colin J Sutherland, Nahla Gadalla, Sharan Atwal, Chris Drakeley, Gibson Kibiki, Teun Bousema, Daniel Chandramohan, Roly Gosling

**Affiliations:** 1 National Institute for Medical Research (NIMR, Mwanza Medical Research Centre, Mwanza, Tanzania; 2 The Global Health Group, University of California, San Francisco, CA, USA; 3 Department of Pediatrics, University of California, San Francisco, CA, USA; 4 Faculty of Infectious and Tropical Diseases, London School of Hygiene and Tropical Medicine, London, UK; 5 Kilimanjaro Clinical Research Institute and Kilimanjaro Christian Medical College, Kilimanjaro Moshi, Tanzania; 6 Department of Medical Microbiology, Radboud University Nijmegen Medical Centre, Nijmegen, The Netherlands

**Keywords:** Spatial Distribution, *Plasmodium Falciparum*, Household Exposure, Parasite Density

## Abstract

**Background:**

At the local level, malaria transmission clusters in hotspots, which may be a group of households that experience higher than average exposure to infectious mosquitoes. Active case detection often relying on rapid diagnostic tests for mass screen and treat campaigns has been proposed as a method to detect and treat individuals in hotspots. Data from a cross-sectional survey conducted in north-western Tanzania were used to examine the spatial distribution of *Plasmodium falciparum* and the relationship between household exposure and parasite density.

**Methods:**

Dried blood spots were collected from consenting individuals from four villages during a survey conducted in 2010. These were analysed by PCR for the presence of *P*. *falciparum*, with the parasite density of positive samples being estimated by quantitative PCR. Household exposure was estimated using the distance-weighted PCR prevalence of infection. Parasite density simulations were used to estimate the proportion of infections that would be treated using a screen and treat approach with rapid diagnostic tests (RDT) compared to targeted mass drug administration (tMDA) and Mass Drug Administration (MDA).

**Results:**

Polymerase chain reaction PCR analysis revealed that of the 3,057 blood samples analysed, 1,078 were positive. Mean distance-weighted PCR prevalence per household was 34.5%. Parasite density was negatively associated with transmission intensity with the odds of an infection being subpatent increasing with household exposure (OR 1.09 per 1% increase in exposure). Parasite density was also related to age, being highest in children five to ten years old and lowest in those > 40 years. Simulations of different tMDA strategies showed that treating all individuals in households where RDT prevalence was above 20% increased the number of infections that would have been treated from 43 to 55%. However, even with this strategy, 45% of infections remained untreated.

**Conclusion:**

The negative relationship between household exposure and parasite density suggests that DNA-based detection of parasites is needed to provide adequate sensitivity in hotspots. Targeting MDA only to households with RDT-positive individuals may allow a larger fraction of infections to be treated. These results suggest that community-wide MDA, instead of screen and treat strategies, may be needed to successfully treat the asymptomatic, subpatent parasite reservoir and reduce transmission in similar settings.

## Background

Malaria transmission is spatially heterogeneous over all geographical scales. At a global level, countries or regions experience varying levels of transmission [1]. Within these regions, transmission is clustered into foci. While the size may vary, the term “focus” is typically used to describe an area of several square kilometres that supports malaria transmission. Within foci, transmission is found to be heterogeneous across smaller units, termed hotspots, which may be a single household or group of households that experience higher than average exposure to infectious mosquitoes [2-4]. While large- and medium-scale patterns of transmission are driven by variations in climate and ecology, the increased risk of exposure observed in hotspots is likely caused by factors such as the proportion of children present, host-genetic polymorphisms, socio-economic status, use of vector control measures, type of housing and micro-environmental factors [5-11]. Evidence suggests that targeting malaria control interventions to hotspots can have a more dramatic impact on transmission than untargeted introduction of control efforts [4,12-14]. Active case detection (ACD), in the form of mass screen and treat (mass blood surveys) campaigns, may be an effective method to detect and treat individuals in hotspots and is being (re)-explored for malaria control and elimination [15-20].

ACD currently relies on rapid diagnostic tests (RDT) or microscopy to identify infected individuals. There is, however, a growing body of evidence that shows that these diagnostic tests miss a substantial proportion of malaria infections in endemic areas compared to PCR[21,22], primarily due to the difficulty of detecting low parasite densities [23-27]. A recent study estimated that in very low prevalence settings, subpatent infections comprise 70-80% of all malaria infections and are responsible for 20-50% of all human-to-mosquito infections [22]. Without treatment, these highly prevalent, low density infections are likely to sustain malaria transmission.

Using aggregated survey prevalence estimates, Okell *et al*. found a positive relationship between transmission and parasite density, with the proportion of infections that are subpatent being highest in low transmission settings [22]. This suggests that at a medium-scale and in high transmission areas, microscopy and RDTs will display adequate sensitivity for the targeting of interventions. It is however, not clear whether this relationship between transmission and parasite density seen in larger geographical areas exists over smaller scales, such as within villages. A better understanding of this issue is important for the detection, and subsequent management, of malaria hotspots.

This study has used intensive cross-sectional sampling in north-western Tanzania to examine household-level heterogeneity in parasite exposure and density. The data obtained were used for simulations of different screen and treat strategies to maximize impact on subpatent malaria infections.

## Methods

### Study site

Misungwi district (latitude 2.85000 S, longitude 33.08333 E) is located in the north-west of Tanzania at an altitude of 1,178 m above sea level. The district has a moderate level of malaria transmission (meso-endemic). The district has two annual rainy seasons, the long rains between February and May and the short rains between November and December. The dry and relatively hot season is June to September. Transmission intensity has a seasonal cycle, with peaks in malaria incidence one to two months after the rains start. The district is situated 60 km from Mwanza town; the prevalence of malaria infection in the region is estimated to be 31.4% by microscopy during a Demographic and Health Survey (DHS).

The population of the district is 308,134, living in 37,468 households, 18.1% of whom are aged five years or under. The district is rural with an economy based around cotton production, rice plantation and fishing from Lake Victoria. The district has a total of 19 wards.

### Sample collection

**Figure 1:**
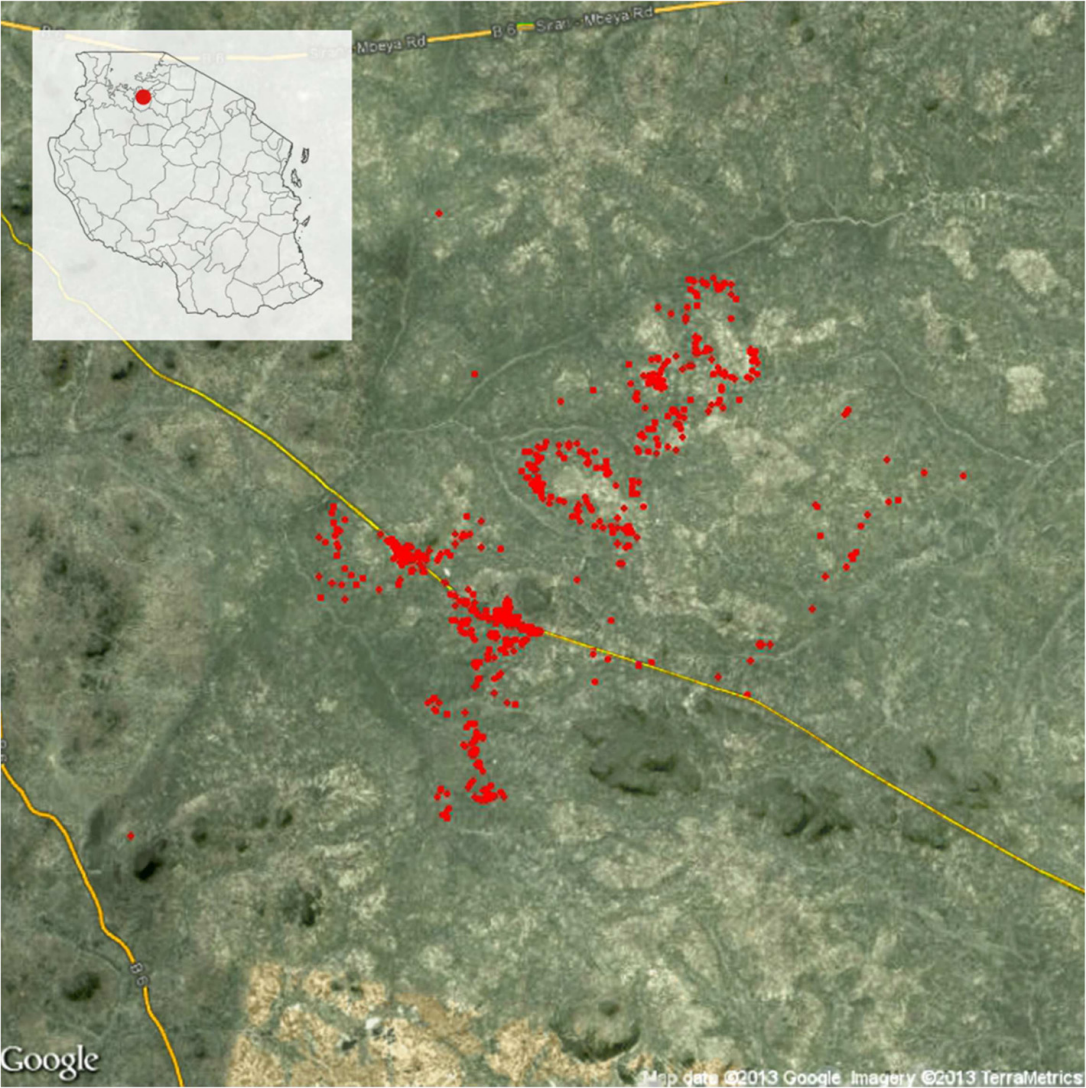
**Location of the study site within Tanzania (inset map) and distribution of households included in the study (red points), showing local topography and road network (yellow lines).**

A census of four villages in a single ward was carried out between August and November 2010 (Figure 1). Every household was visited and mapped by GPS. All individuals in the ward were invited to participate in the study. For those who were not present, the head of household gave information on their age, sex and ITN use. Every household was visited and mapped by GPS. Individuals who consented to join the study were asked to provide a finger-prick sample of blood into Whatman® standard 3mm filter paper for parasite detection and had their temperature measured by digital thermometer. Any subject who reported a fever within the previous 24 hours was tested for malaria using a histidine-rich protein 2 (HRP2) rapid malaria diagnostic test (RDT, *Paracheck*-*Pf*®, Orchid Biomedical Systems, Goa, India) and referred to a study clinician for management of their febrile illness. Filter papers were dried overnight and stored individually with desiccant at −20°C for later molecular analysis.

### Molecular estimation of parasite prevalence and density

DNA was extracted from filter papers using the Chelex®(Sigma, USA) extraction method described previously [28] in 96-deep well plates. Parasite DNA was detected using nested PCR (nPCR) targeting the 18S rRNA gene as previously described [29]. Parasite density was estimated for all positive PCR samples using a quantitative PCR (qPCR) using the methods of Beshir *et al*. [30] with the following modifications. Duplex 10 mL reactions amplifying both human (b-tubulin) and *Plasmodium* (Met tRNA gene) targets were run in duplicate for each sample in a 354-well format ABI qPCR machine, model 7500. DC_T_ values between the two targets were estimated for each sample and the mean DC_T_ of duplicate wells normalized to the within-run quantitative standard, comprising the WHO International Standard for *Plasmodium falciparum* DNA (IS) [28,31], and representing 500 parasites mL^-1^. The ratio of parasite density in the sample relative to the IS was then multiplied by 500 parasites mL^-1^ to obtain the estimate of parasite density. Any samples that were negative by qPCR but positive by nPCR were assigned an arbitrary parasite density value of half the minimum density detected.

### Household exposure

Household exposure to malaria infection was estimated using distance weighted local prevalence of malaria infection (detected by PCR) [32]. This method calculates parasite prevalence amongst all neighbouring households within 1 km of the index house, weighting the prevalence estimate according to the inverse of the distance of neighbouring households to the index. Olotu *et al*. showed that this method provides a suitable index for exposure, as it was predictive of individual infection in the index household [32].

### Modelling relationship between household exposure and parasite density

Due to the distribution of the parasite density data, parasite density was modelled as a binary outcome; subpatent (> 0 and <100 parasites/μl) 1; patent (>100 parasites/μl) 0. This classification was based on the density typically quoted as the detection limit for RDTs [33]. Uninfected individuals were not included in the analysis. Both household exposure and individual age were explored as explanatory variables in univariate logistic regression. Variables were retained in a multivariate model if they were significant at the 10% level. To explore a possible non-linear relationship with household exposure and age, these variables were also categorized. Household exposure was split into quartiles based on the distribution of distance-weighted PCR prevalence: < 26.3%; 26.4-31.7%; 31.8-39.8%; and > 39.8%. Age in years was categorized into five groups: < 5; 5–10; 11–20; 21–40; and > 40 years. Model fit using categorized *versus* continuous values of household exposure and age was compared using Akaike Information Criterion (AIC) values and likelihood ratio tests.

### Simulating different screen and treat approaches

In order to estimate the number of infections that would be detected by RDT and treated accordingly, and to explore whether it was possible to increase the proportion of PCR positives that would be correctly treated using RDTs, simulations of targeted mass drug administration (tMDA) were conducted. This system works by first defining a threshold household prevalence. In households where the prevalence of infection by RDT exceeds this threshold, all individuals in that household are treated irrespective of their RDT result. For example, if the threshold was set at 50%, all individuals in households where at least 50% tested positive by RDT would be treated. Simulations using thresholds of 10%-100%, in increments of 10%, were conducted. Using the parasite density estimates obtained from the qPCR, individuals were assumed to test positive to an RDT if they had a parasite density of >100 parasites/μl. Simulations using detection limits of 50 parasites/μl and 200 parasites/μl were also run for comparison. These represent a more ideal detection limit and the lowest density used by WHO in quality control tests respectively [34]. This simple cutoff approach was used as opposed to modelling the relationship between PCR and the RDT data, as RDTs were only used on individuals with reported recent fever.

## Results

### PCR data

The census revealed that approximately 3,800 individuals lived in the 4 study villages. Dried blood spots were collected from 3,057 individuals (80.4%) and 52.7% of participants were male. Overall prevalence of infection by nPCR (two rounds of amplification) was 35.2%. The single round qPCR was less sensitive, as expected, such that 601 of the 1,078 nPCR positives were negative by qPCR. For the purpose of further analysis and simulations, these were assigned a density value of half the minimum density (eight parasites/μl) of the 477 samples that tested positive by qPCR. Having corrected for qPCR negative/nPCR positive samples, geometric mean density of infection was 153 parasites/μl (range 8–532,001 parasites/μl); 56.2% of infections (n = 606) were of a density <100 parasites/μl.

### Household exposure

**Figure 2:**
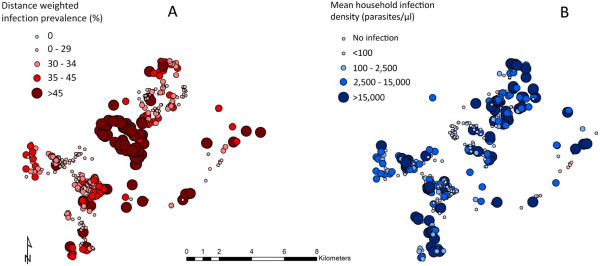
**Micro**-**epidemiology of infection in the study region. A** - Household exposure estimated using distance-weighted PCR prevalence with a 1-km window. **B** - mean household parasite density (infected individuals only).

Mean household exposure as estimated by distance-weighted nPCR prevalence was 34.5% (range 0–94.7%). Figure 2A, which shows exposure for each household in the study site, illustrates that exposure was spatially heterogeneous with highest exposure households clustering in a central hotspot in the study area.

### Density of infection in relation to exposure and age

**Figure 3:**
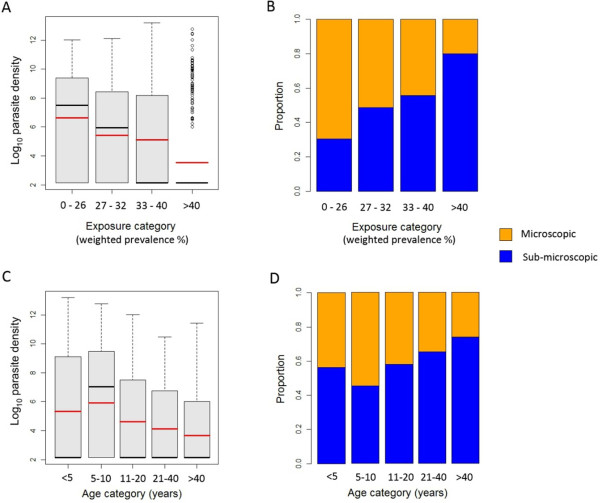
**Relationship between parasite density and household exposure. A** – Boxplot of log transformed parasite densities over different exposure categories (based on quintiles). Black lines indicate median values, red lines indicate mean values. **B** – The proportion of subpatent (<100 parasites/μl) infections over different exposure categories. **C** – Boxplot of log transformed parasite densities over different age categories. **D** – The proportion of subpatent (<100 parasites/μl) infections over different age categories.

Parasite infection densities displayed a negative spatial relationship with exposure, with mean infection densities being lowest in the highest exposure households and *vice versa* (Figure 2A and 2B). Figure 3A shows the relationship between household exposure and parasite density, suggesting that infection densities decreased with increasing household exposure. This was reflected in the distribution of infections below 100 parasites/μl (hereafter “subpatent”) across exposure categories (Figure 3B), with 30.5% of infections being subpatent in the lowest exposure households and 79.9% of infections being subpatent in the highest exposure households. Figure 3C shows the relationship between density of infection and age group, which suggests a non-linear relationship. This was reflected in the distribution of subpatent infection across age groups (Figure 3D), with the proportion of infections being subpatent lowest in the five to ten year olds (45.4%) and highest in those > 40 years old (79.9%).

### Modelling parasite density

Results of univariate logistic regression supported the observed positive association between household exposure and subpatent infection (OR 1.08, 95% CI 1.06-1.1 p < 0.001) (Table 1). AIC values and a likelihood ratio test suggested that including household exposure as a linear predictor provided a better model fit than including exposure as a categorical variable. Including age as a categorical variable provided a better model fit than when age was included as a continuous variable, with the odds of an infection being subpatent being lower in five to ten year olds (OR 0.52, p < 0.01) and higher in > 40 year olds (OR 3.03, p < 0.001) compared to under five year olds. When included together, both household exposure and age group remained significant predictors, showing similar relationships to those found with univariate regression (Table 1).

**Table 1:** Results of the univariate and multivariate logistic regression of determinants of parasite density

**Variable**		**Univariate**	**Multivariate**
	**Number sub**-**patent / Number infected (%)**	**OR 95% CI p**-**value**	**OR 95% CI p-value**
Household exposure (%)		1.08* 1.06-1.1 <0.001	1.09* 1.07-1.11 <0.001
Age group (years)			
<5	128 / 227 (56.4%)	1	1
5-10	163 / 359 (45.4%)	0.52 0.33-0.82 <0.001	0.58 0.37-0.91 0.02
11-20	141 / 244 (57.8%)	1.03 0.63-1.7 0.89	1.18 0.71-1.94 0.51
21-40	69 / 106 (65.1%)	1.65 0.88-3.09 0.12	1.77 0.94-3.32 0.08
>40	105 / 142 (73.9%)	3.03 1.65-5.59 <0.001	3.46 1.87-6.37 <0.001

Parasite density was modelled as a binary outcome; sub-patent (> 0 and <100 parasites/μl) or patent (>100 parasites/μl).

*Per percentage point.

### Simulation of different treatment approaches

**Figure 4:**
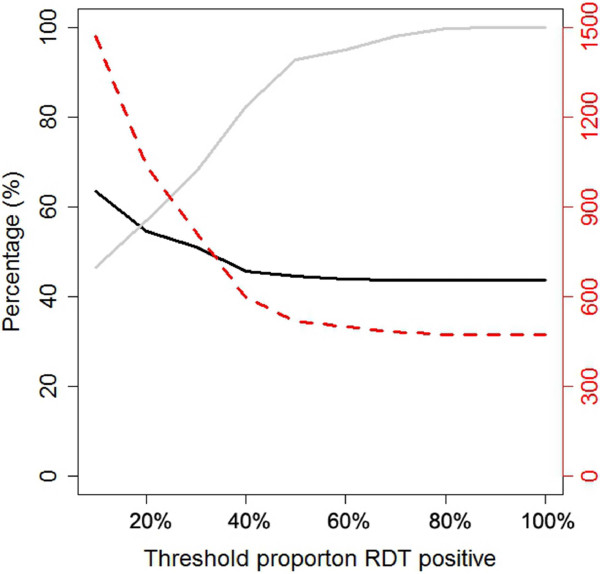
**Results of the tMDA simulations assuming different intervention thresholds.** The black line represents the percentage of infections that would be correctly treated. The grey line represents the percentage of treatments that would be correctly administered to true positives. The red dashed line indicates the number of treatments that would be administered.

Simulations of treatment decisions using different household prevalence thresholds to trigger household delivery of tMDA showed that, as expected, decreasing the threshold led to an increase in the proportion of infections that would be treated (Figure 4). For example, if a threshold of 0.2 was used, the proportion of infections treated increased from 43 to 55%. However, this led to a corresponding increase in the number of treatments administered from 472 (15.4% of the population) to 1,035 (33.9% of the population), with a decrease in the proportion of treatments that would be correctly administered from 100 to 43% (Figure 4). Results were similar if the detection limit for RDT was assumed to be 50 or 200 parasites/μl.

## Discussion

This paper investigates the micro-epidemiology of *P*. *falciparum* infection in a rural community in north-western Tanzania, exploring the relationship between parasite density and exposure (distance-weighted parasite prevalence), using a novel application of qPCR on DNA from filter-paper blood-spots. In contrast to studies conducted over larger geographical areas, this study found that in a moderate transmission setting, at the household level, the proportion of infections defined as subpatent is positively associated with exposure. If similar findings are found in other settings, this suggests that microscopy and RDTs are unlikely to have adequate sensitivity to identify transmission hotspots where acquired immunity allows people to control infections to very low densities; yet these are the exact places one needs to detect cases during a mass screen and treat campaign. Simulations showed that the proportion of infections that are correctly identified and treated can be increased using tMDA. However, this approach still misses a large proportion of infections, suggesting that MDA of entire foci may be required for interruption of transmission.

Results of qPCR analysis showed that 56.2% of infections were of a density <100 parasites/μl, the density typically quoted as the limit of detection for routine microscopy and RDTs. This finding fits with the analysis of Okell *et al*. who estimated that where prevalence of infection is 35% by PCR, around 60% of infections are missed by microscopy [22]. This study found that, controlling for age, parasite density showed a negative relationship with exposure. Such a finding is supported by Clarke *et al*., who showed that with increasing proximity to the River Gambia, the prevalence of infection increased but the density of infection decreased [35]. These micro-epide-miological patterns are markedly different from patterns between endemic regions where lower endemicity is related to lower average parasite density and a larger proportion of infections that are below the microscopic threshold for detection [22].

Multiple factors can influence the relationship between parasite exposure and the density of blood stage infection, and these factors may be more or less apparent at different spatial scales. Potential explanations for a negative association between exposure and parasite density within a focus, seen in this study at a microscale, is that highly exposed individuals acquire greater blood stage immunity, controlling parasite densities more effectively [36]. On the other hand, an explanation for a positive association between exposure and parasite density across different foci, as simulated by Arnot *et al*. [30] is that differences in the age of infection may over-ride the influence of exposure-related immunity. In addition, it is likely that parasite diversity is lower in foci of lower transmission, potentially enhancing the acquisition of immunity to circulating strains [30,37].

If, over small geographical regions, density of infection is indeed lowest among those at highest exposure, there are substantial implications for mass screen and treat campaigns that plan to rely on RDTs for diagnosis. As this data suggest, due to a positive association between RDT sensitivity and parasite density, RDTs are likely to display lowest sensitivity in transmission hotspots. Failure to properly target individuals in hotspots may allow transmission to persist. Use of more sensitive diagnostics might be able to circumvent this problem. However, those currently available, such as PCR and LAMP, are not yet practical for widespread routine field use (Gadalla and Mosha, in prep), and point of care serological tests, that may be able to differentiate hotspot households, remain in development. An alternative approach, extrapolating from qPCR data of this study, is to target MDA to those households with highest prevalence of infection by RDT. The simulations show that such an approach increases the proportions of true positives who would be treated. However, even with a threshold of 10% prevalence of RDT positives in a household, where nearly half the population would be treated, an estimated 36.5% of infections would still go untreated.

The importance of the subpatent asymptomatic parasite pool rests on the understanding that subpatent infection can transmit malaria. While subpatent infections are known to transmit infection [38,39], the absolute density of infection necessary to transmit is unknown. nPCR is typically able to detect infections down to approximately one parasites/μl using dried blood spots, which equates to approximately 5,000,000 parasites in an adult. Presumably there are many infections below this threshold that were not detected. Whether to screen and treat using a more sensitive tool or to institute MDA without targeting requires a better understanding of the minimum density of malaria parasites that result in human-mosquito transmission, as well as the comparative costs and operational ease of different approaches.

This study has several limitations. Firstly, the qPCR method applied here is novel, as this is the first study to use qPCR on filter-paper blood-spot DNA to provide an estimate of parasite density in a cross-sectional survey. This was achieved without having to measure the volume of blood used in the assay. This method is yet to be fully validated on filter-paper samples from the field; further, the parasite target used is pangenus, and some contribution from *Plasmodium malariae* and *Plasmodium ovale* spp. to the density estimates cannot be ruled out [24,30]. Secondly, RDTs do not have a clear-cut detection limit but show a smoother decline in sensitivity as parasite density drops. Reliable, randomly sampled RDT data were, however, not available, as RDTs were only used on individuals with reported recent fever. The use of a simple RDT detection limit (100mL^-1^) may overestimate the numbers of infections that would be missed, but would not change the overall finding that RDT sensitivity is likely to be least adequate in hotspot households. Thirdly, it is possible that PCR produced some false negatives and false positives which could affect conclusions. That said, false negatives would presumably be due to very low density infections which likely play a minor role in transmission. False positives are unlikely, but may have arisen due to contamination, although all steps were taken to minimize this possibility. Lastly, assumptions were made that, distance-weighted PCR prevalence provides a suitable estimate of exposure and location of hotspots. This assumption is based on the study by Olotu *et al*. [32], which showed that distance-weighted prevalence was a good predictor of household infection. It would be interesting to explore whether these findings hold using alternative methods to measure exposure, such as Entomological Inoculation Rate (EIR), but it was not possible to make these measurements in this study. Assuming transmission hotspots are stable over time, serology may also help to identify hotspot households.

## Conclusion

This study has examined the micro-epidemiology of malaria, exploring the spatial relationship between parasite prevalence and density using molecular methods of detection, in a site with moderate transmission of *P*. *falciparum* in Tanzania. The study found a negative relationship between density of infection and exposure, with the proportion of subpatent infections increasing with increasing exposure. Simulations of different tMDA strategies suggest that treating all individuals in households where RDT prevalence was above 20% would increase the number of infections treated from 43 to 55%. However, 45% of infections would remain untreated, suggesting that community-wide MDA may be needed to successfully treat the asymptomatic parasite reservoir in communities such as the one studied.

## Competing interests

The authors declare that they have no competing interests.

## Authors’ contributions

JFM was involved in the study design, supervised the implementation of the study and data collection, analysed data, drafted and revised the manuscript. HJWS and BG were involved in data analysis, interpretation of the data, drafted and revised the manuscript. DC and RG were involved in overall study design and supervision, interpretation of the data and revisions of the manuscript. TB, CS and CD were involved in supervision of laboratory work, interpretation of the data and revision of the manuscript. BG and GK were involved in interpretation of the data and revisions of the manuscript. NG and SA performed the real-time PCR testing and revised the manuscript. All authors have read and approved the final version of the manuscript.

## References

[B1] Gething PW Patil AP Smith DL Guerra CA Elyazar IRF Johnston GL Tatem AJ Hay SI A new world malaria map: *Plasmodium falciparum* endemicity in 2010 Malar J 2011 10 378 10.1186/1475-2875-10-378 22185615 PMC3274487

[B2] Oesterholt MJAM Bousema JT Mwerinde OK Harris C Lushino P Masokoto A Mwerinde H Mosha FW Drakeley CJ Spatial and temporal variation in malaria transmission in a low endemicity area in northern Tanzania Malar J 2006 5 98 10.1186/1475-2875-5-98 17081311 PMC1635725

[B3] Clark TD Greenhouse B Njama-Meya D Nzarubara B Maiteki-Sebuguzi C Staedke SG Seto E Kamya MR Rosenthal PJ Dorsey G Factors determining the heterogeneity of malaria incidence in children in Kampala, Uganda J Infect Dis 2008 198 393 400 10.1086/589778 18522503

[B4] Bousema T Griffin JT Sauerwein RW Smith DL Churcher TS Takken W Ghani A Drakeley C Gosling R Hitting hotspots: spatial targeting of malaria for control and elimination PLoS Med 2012 9 e1001165 10.1371/journal.pmed.1001165 22303287 PMC3269430

[B5] Gamage-Mendis AC Carter R Mendis C De Zoysa AP Herath PR Mendis KN Clustering of malaria infections within an endemic population: risk of malaria associated with the type of housing construction AmJTrop Med Hyg 1991 45 77 85 10.4269/ajtmh.1991.45.77 1867350

[B6] Alonso PL Lindsay SW Armstrong Schellenberg JR Keita K Gomez P Shenton FC Hill AG David PH Fegan G Cham K A malaria control trial using insecticide-treated bed nets and targeted chemoprophylaxis in a rural area of The Gambia, West Africa. 6. The impact of the interventions on mortality and morbidity from malaria Trans R Soc Trop Med Hyg 1993 87 Suppl 2 37 44 8212109 10.1016/0035-9203(93)90174-o

[B7] Ghebreyesus TA Haile M Witten KH Getachew A Yohannes M Lindsay SW Byass P Household risk factors for malaria among children in the Ethiopian highlands Trans R Soc Trop Med Hyg 2000 94 17 21 10.1016/S0035-9203(00)90424-3 10748890

[B8] ter Kuile FO Terlouw DJ Phillips-Howard PA Hawley WA Friedman JF Kolczak MS Kariuki SK Shi YP Kwena AM Vulule JM Nahlen BL Impact of permethrin-treated bed nets on malaria and all-cause morbidity in young children in an area of intense perennial malaria transmission in western Kenya: cross-sectional survey AmJTrop Med Hyg 2003 68 100 107 12749492

[B9] Brooker S Clarke S Njagi JK Polack S Mugo B Estambale B Muchiri E Magnussen P Cox J Spatial clustering of malaria and associated risk factors during an epidemic in a highland area of western Kenya Trop Med Int Health 2004 9 757 766 10.1111/j.1365-3156.2004.01272.x 15228485

[B10] Bodker R Msangeni HA Kisinza W Lindsay SW Relationship between the intensity of exposure to malaria parasites and infection in the Usambara Mountains, Tanzania AmJTrop Med Hyg 2006 74 716 723 16687668

[B11] Williams TN Human red blood cell polymorphisms and malaria Curr Opin Microbiol 2006 9 388 394 10.1016/j.mib.2006.06.009 16815736

[B12] Bejon P Williams TN Liljander A Noor AM Wambua J Ogada E Olotu A Osier FHA Hay SI Farnert A Marsh K Stable and unstable malaria hotspots in longitudinal cohort studies in Kenya PLoS Med 2010 7 e1000304 10.1371/journal.pmed.1000304 20625549 PMC2897769

[B13] Zhou G Githeko AK Minakawa N Yan G Community-wide benefits of targeted indoor residual spray for malaria control in the western Kenya highland Malar J 2010 9 67 10.1186/1475-2875-9-67 20199674 PMC2843726

[B14] Githeko AK Ototo EN Guiyun Y Progress towards understanding the ecology and epidemiology of malaria in the western Kenya highlands: opportunities and challenges for control under climate change risk Acta Trop 2012 121 19 25 10.1016/j.actatropica.2011.10.002 22015426 PMC3298846

[B15] Utarini A Chandramohan D Nystrom L Comparison of active and passive case detection systems in Jepara District, Indonesia Asia Pac J Public Health 2007 19 14 17 10.1177/10105395070190010401 17784654

[B16] Lee P-W Liu C-T Rampao HS do Rosario VE Shaio M-F Pre-elimination of malaria on the island of Principe Malar J 2010 9 26 10.1186/1475-2875-9-26 20089158 PMC2823607

[B17] Stresman GH Kamanga A Moono P Hamapumbu H Mharakurwa S Kobayashi T Moss WJ Shiff C A method of active case detection to target reservoirs of asymptomatic malaria and gametocyte carriers in a rural area in Southern Province, Zambia Malar J 2010 9 265 20920328 10.1186/1475-2875-9-265 PMC2959066

[B18] Herdiana H Fuad A Asih PB Zubaedah S Arisanti RR Syafruddin D Kusnanto H Sumiwi ME Yuniarti T Imran A Rahmadyani R Yani M Kusriastuti R Tarmizi SN Laihad FJ Hawley WA Progress towards malaria elimination in Sabang Municipality, Aceh, Indonesia Malar J 2013 12 42 10.1186/1475-2875-12-42 23363768 PMC3564785

[B19] Sturrock HJW Hsiang MS Cohen JM Smith DL Greenhouse B Bousema JT Gosling RD Targeting asymptomatic malaria infections: active surveillance in control and elimination PLoS Med 2013 10 e1001467 10.1371/journal.pmed.1001467 23853551 PMC3708701

[B20] Sturrock HJW Novotny JM Kunene S Dlamini S Zulu Z Cohen JM Hsiang MS Greenhouse B Gosling RD Reactive case detection for malaria elimination: real-life experience from an ongoing program in Swaziland PLoS One 2013 8 e63830 10.1371/journal.pone.0063830 23700437 PMC3658965

[B21] Okell LC Ghani AC Lyons E Drakeley CJ Submicroscopic infection in *Plasmodium falciparum*-endemic populations: a systematic review and meta-analysis J Infect Dis 2009 200 1509 1517 10.1086/644781 19848588

[B22] Okell LC Bousema T Griffin JT Ouedraogo AL Ghani AC Drakeley CJ Factors determining the occurrence of submicroscopic malaria infections and their relevance for control Nat Comm 2012 3 1237 10.1038/ncomms2241 PMC3535331 23212366

[B23] Banchongaksorn T Prajakwong S Rooney W Vickers P Operational trial of ParaSight-F (dipstick) in the diagnosis of falciparum malaria at the primary health care level Southeast Asian J Trop Med Public Health 1997 28 243 246 9444000

[B24] Bojang KA The diagnosis of *Plasmodium falciparum* infection in Gambian children, by field staff using the rapid, manual, ParaSight-F test Ann Trop Med Parasitol 1999 93 685 687 10.1080/00034989957925 10715695

[B25] Stow NW Torrens JK Walker J An assessment of the accuracy of clinical diagnosis, local microscopy and a rapid immunochromatographic card test in comparison with expert microscopy in the diagnosis of malaria in rural Kenya Trans R Soc Trop Med Hyg 1999 93 519 520 10.1016/S0035-9203(99)90359-0 10696409

[B26] Tham JM Lee SH Tan TM Ting RC Kara UA Detection and species determination of malaria parasites by PCR: comparison with microscopy and with ParaSight-F and ICT malaria Pf tests in a clinical environment J Clin Microbiol 1999 37 1269 1273 10203469 10.1128/jcm.37.5.1269-1273.1999 PMC84748

[B27] van den Broek I Hill O Gordillo F Angarita B Hamade P Counihan H Guthmann J-P Evaluation of three rapid tests for diagnosis of *P*. *falciparum* and *P*. *vivax* malaria in Colombia AmJTrop Med Hyg 2006 75 1209 1215 17172395

[B28] Plowe CV Djimde A Bouare M Doumbo O Wellems TE Pyrimethamine and proguanil resistance-conferring mutations in *Plasmodium falciparum* dihydrofolate reductase: polymerase chain reaction methods for surveillance in Africa AmJTrop Med Hyg 1995 52 565 568 10.4269/ajtmh.1995.52.565 7611566

[B29] Snounou G Singh B Nested PCR analysis of Plasmodium parasites Methods Mol Med 2002 72 189 203 12125116 10.1385/1-59259-271-6:189

[B30] Arnot D Unstable malaria in Sudan: the influence of the dry season. Clone multiplicity of *Plasmodium falciparum* infections in individuals exposed to variable levels of disease transmission Trans R Soc Trop Med Hyg 1998 92 580 585 10.1016/S0035-9203(98)90773-8 10326095

[B31] Padley DJ Heath AB Sutherland C Chiodini PL Baylis SA Collaborative Study G Establishment of the 1st World Health Organization International Standard for *Plasmodium falciparum* DNA for nucleic acid amplification technique (NAT)-based assays Malar J 2008 7 139 10.1186/1475-2875-7-139 18652656 PMC2518157

[B32] Olotu A Fegan G Wambua J Nyangweso G Ogada E Drakeley C Marsh K Bejon P Estimating individual exposure to malaria using local prevalence of malaria infection in the field PLoS One 2012 7 e32929 10.1371/journal.pone.0032929 22479349 PMC3315550

[B33] Ochola LB Vounatsou P Smith T Mabaso ML Newton CR The reliability of diagnostic techniques in the diagnosis and management of malaria in the absence of a gold standard Lancet Infect Dis 2006 6 582 588 10.1016/S1473-3099(06)70579-5 16931409

[B34] FIND Malaria rapid diagnostic test performance 2012 Geneva, Switzerland: Results of WHO product testing of malaria RDTs: Round 4

[B35] Clarke SE Bogh C Brown RC Walraven GEL Thomas CJ Lindsay SW Risk of malaria attacks in Gambian children is greater away from malaria vector breeding sites Trans R Soc Trop Med Hyg 2002 96 499 506 10.1016/S0035-9203(02)90419-0 12474476

[B36] Greenhouse B Slater M Njama-Meya D Nzarubara B Maiteki-Sebuguzi C Clark TD Staedke SG Kamya MR Hubbard A Rosenthal PJ Dorsey G Decreasing efficacy of antimalarial combination therapy in Uganda is explained by decreasing host immunity rather than increasing drug resistance J Infect Dis 2009 199 758 765 10.1086/596741 19199542 PMC2733854

[B37] Konate L Zwetyenga J Rogier C Bischoff E Fontenille D Tall A Spiegel A Trape JF Mercereau-Puijalon O Variation of *Plasmodium falciparum* msp1 block 2 and msp2 allele prevalence and of infection complexity in two neighbouring Senegalese villages with different transmission conditions Trans R Soc Trop Med Hyg 1999 93 Suppl 1 21 28 10450422 10.1016/s0035-9203(99)90323-1

[B38] Harris I Sharrock WW Bain LM Gray K-A Bobogare A Boaz L Lilley K Krause D Vallely A Johnson M-L Gatton ML Shanks GD Cheng Q A large proportion of asymptomatic Plasmodium infections with low and sub-microscopic parasite densities in the low transmission setting of Temotu Province, Solomon Islands: challenges for malaria diagnostics in an elimination setting Malar J 2010 9 254 10.1186/1475-2875-9-254 20822506 PMC2944341

[B39] Manjurano A Okell L Lukindo T Reyburn H Olomi R Roper C Clark TG Joseph S Riley EM Drakeley C Association of sub-microscopic malaria parasite carriage with transmission intensity in north-eastern Tanzania Malar J 2011 10 370 10.1186/1475-2875-10-370 22177014 PMC3276450

